# Periodic Active Case Finding for TB: When to Look?

**DOI:** 10.1371/journal.pone.0029130

**Published:** 2011-12-22

**Authors:** Peter J. Dodd, Richard G. White, Elizabeth L. Corbett

**Affiliations:** 1 London School of Hygiene and Tropical Medicine, London, United Kingdom; 2 Malawi-Liverpool Wellcome Trust Clinical Research Programme, Blantyre, Malawi; National AIDS Research Institute, India

## Abstract

**Objective:**

To investigate the factors influencing the performance and cost-efficacy of periodic rounds of active case finding (ACF) for TB.

**Methods:**

A mathematical model of TB dynamics and periodic ACF (PACF) in the HIV era, simplified by assuming constant prevalence of latent TB infection, is analyzed for features that control intervention outcome, measured as cases averted and cases found. Explanatory variables include baseline TB incidence, interval between PACF rounds, and different routine and PACF case-detection rates among HIV-infected and uninfected TB cases.

**Findings:**

PACF can be cost-saving over a 10 year time frame if the cost-per-round is lower than a threshold proportional to initial incidence and cost-per-case-treated. More cases are averted at higher baseline incidence rates, when more potent PACF strategies are used, intervals between PACF rounds are shorter, and when the ratio of HIV-negative to positive TB cases detected is higher. More costly approaches, e.g. radiographic screening, can be as cost-effective as less costly alternatives if PACF case-detection is higher and/or implementation less frequent.

**Conclusion:**

Periodic ACF can both improve control and save medium-term health care costs in high TB burden settings. Greater costs of highly effective PACF at frequent (e.g. yearly) intervals may be offset by higher numbers of cases averted in populations with high baseline TB incidence, higher prevalence of HIV-uninfected cases, higher costs per-case-treated, and more effective routine case-detection. Less intensive approaches may still be cost-neutral or cost-saving in populations lacking one or more of these key determinants.

## Introduction

Tuberculosis (TB) continues to pose a major global health problem, causing an estimated 9.4 million new cases and 1.7 million deaths during 2009 [1]. TB is infectious and treatable, and therefore identifying, diagnosing and starting active cases on appropriate medication is at the core of current policies for TB control [2]. Modelling work [3]–[6] has repeatedly emphasized the importance of case detection. Infrastructure is poor in many of the settings where TB incidence is at its highest [7], [8], and medium term methods for improving case acquisition and treatment will be needed to hit the STOP TB goals for 2015. Interventions such as active case finding (ACF) that screen large portions of the population for active disease have a long history in TB control [9]. Recent work has successfully demonstrated the potential of ACF to reduce TB prevalence in contemporary settings with high HIV prevalence [10], and intensified interest in the possibilities for scale-up [11].

The sorts of control effort categorized as ACF are usually too intensive to be continuously sustainable in resource-poor communities. In these cases, the intervention is typically applied as a series of rounds: periodic active case finding (PACF). The choice of intervention period affects the natural epidemic dynamics, which rebound between rounds; and has the potential to differently affect the case ascertainment of HIV-infected and HIV-uninfected TB cases, whose contrasting typical duration affects their chances of falling between rounds. This raises a series of questions around the effect of round frequency on intervention outcomes and cost, which have been subject to very few direct investigations [12].

In this paper, we employ a simple mathematical model of TB transmission and disease among individuals who may be infected with HIV. The model is parametrized by the initial TB incidences and disease durations (different by HIV infection status), the distribution of delays from recent infection to disease, and the proportion of disease that is due to recent infection. We explore the effect of community and intervention characteristics on outcomes such as cases averted, cases found, and the total cost, over a 10 year time-frame.

## Methods

### General model: incidence from prevalence

The usual assumption is that upon infection with *Mycobacterium tuberculosis* (MTB) for the first time, there is a chance 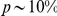 that the individual will develop active TB disease within around 5 years [13]–[15]. For the remaining 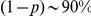 of people, the risk of TB has been estimated to be much lower, e.g. 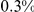 per year [15], though this may vary by population [16]. This situation can be captured by assuming a mixture model for the probability of having avoided disease for a time 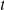 after infection (i.e. the associated survival function), 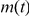:
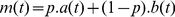
(1)N.B. ‘survival’ in this general terminology refers to avoiding a specified event, which need not be death. The risk for the ‘fast’ route derived in [15] is well fit by an exponential survival function with rate 0.9 per year. We shall write 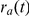, 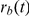 and 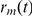 for the hazards associated with survival from disease via the: fast route (survival function 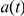); slow route (survival function 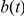); and either route (survival function 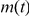), respectively.

For those who have already been infected, it is often assumed that they have some protection from progression to disease. We will denote the factor reduction in likelihood of progression 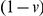. The value of protection 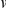 is not well known, but has been estimated, e.g., as 16–41% in [15], and 63% for men and 81% for women in [14].

Let us denote by 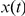 and 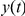 the fractions of the population who are uninfected and infected by MTB at time 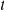, respectively; and write 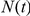 for the population size and 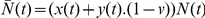 for an effectively susceptible population. If the proportion of those remaining alive at time 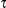 after their infection by TB is 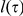, then, ignoring the fraction of the population with TB disease, the incidence at time 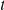 is
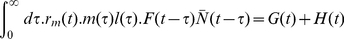
(2)where 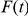 is the force-of-infection at time 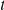, and where 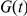 and 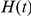 are
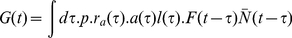
(3)

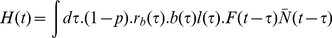
(4)Equation 2 represents the current incidence as the result of the history of infection combined with delays to disease.

The proportion of incident cases at time 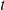 that is ‘recent’ is
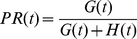
(5)


### Approximations

In this section we will justify two simplifying approximations to this model, namely: that the prevalence of latent TB infection is roughly constant; and that the incidence, 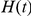, due to distant infection is roughly constant. These approximations are reasonable over the time frames considered (10 years or less), but break down for longer time frames.

In a (possibly growing) population, with annual risk of MTB infection 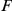, simple manipulation of the differential equations for the total number, number infected, and number uninfected shows that the fraction of the population infected grows with rate 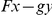, where 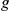 is the per-capita birth-rate that dilutes the infected pool. This means, starting with equilibrium values 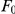, 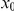 and 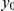 holding at time 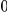, 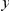 satisfies
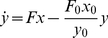
(6)If the risk of infection has changed to a new constant 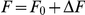, while time is such that 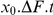 is small compared with 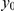, Equation 6 has the approximate solution
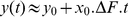
(7)In a high burden setting, 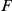 is typically less than a few percent, and 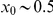. Thus 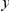 is likely to change at no more than 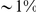 per year, e.g. [17], [18].

For a constant population, the above approximation means that 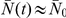, and by linearity, 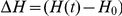 obeys:
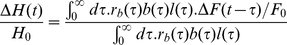
(8)

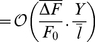
(9)where 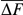 is the mean change in the force-of-infection over the duration of intervention, 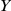 denotes the duration of intervention, and 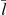 is the average life-span after infection. This follows since the rate of slow TB progression is negligible compared to mortality.

Assuming that 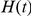 is approximately constant is therefore a reasonable approximation over time frames shorter than a life-span.

### Simplified model

With the approximation that 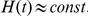, we can write the incidence in terms of the baseline proportion of TB that is ‘recent’, 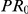, as
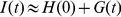
(10)

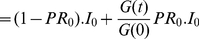
(11)

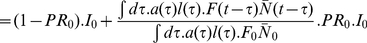
(12)The last equality, resulting in Equation 12, follows because we further assume that the hazard, 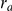, associated with the ‘recent’ route is constant, and since all constant factors in the numerator 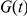 cancel with those in the denominator 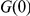.

We will restrict ourselves to the situation where the population size is roughly constant, which, together with the approximation that 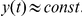, and neglecting mortality over the ‘fast’ timescale, means that
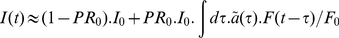
(13)where 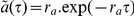 is the distribution of (fast) delays from infection to disease. This represents a risk of developing TB disease that is highest immediately after infection, decaying towards a steady background rate in subsequent years. It will be convenient to use the notation
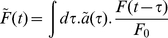
(14)We will make the usual mass-action assumption that 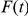 is proportional to the number of active TB cases, 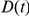, so that
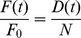
(15)Together, Equation 13 and Equation 15 form a dynamical rule for relating incidence to the prevalence of active TB cases. (A rule for calculating 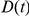 from incidence, thus specifying a complete dynamics, is provided in the section after next.) Note that the uncertain parameters for the endogenous reactivation rate and the protection from disease due to previous infection are not needed.

### The influence of HIV

In many sub-Saharan African settings, a substantial proportion of incident TB disease cases are HIV-infected [1]. Assuming a constant underlying HIV prevalence and a constant incidence rate ratio (IRR) for developing TB if infected with HIV, we can determine the incidence of HIV-infected and HIV-uninfected TB cases in the general population 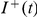, 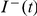, as
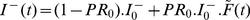
(16)

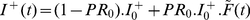
(17)where, again, zero subscripts indicate equilibrium values at time 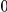. Note, we are assuming that the IRR applies equally to individuals progressing via the ‘fast’ and ‘slow’ routes.

In the presence of HIV, we again assume that 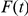 is proportional to the prevalence of active TB cases, but with a discount factor 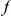 for HIV-infected TB cases to capture their reduced likelihood of being smear positive [19]. Writing 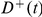 and 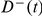 for the number of HIV-infected and HIV-uninfected cases of TB, this means taking
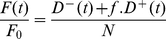
(18)


### Prevalence from incidence

This section applies to the prevalences and incidences of both HIV-infected and HIV-uninfected TB disease. Since the form of each set of equations is identical, we drop superscript 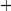 and 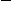 signs and refer to a generic prevalence and incidence. The evolution of the density of active TB cases, 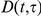, who have been active for a time 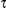 at time 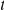 is modelled by the partial differential equation and boundary condition:
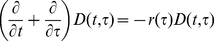
(19)

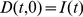
(20)where 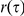 is the total removal rate (due to detection, death and self-cure), and 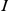 is the incidence. This can be solved by characteristics to give
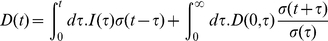
(21)where 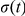 is the survival function for a case remaining active and undetected until time 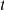, generated by the hazard 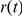, and 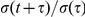 is the conditional survival given a case that has already remained active for a time 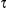. If the system has been at equilibrium until time 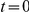, then 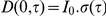 and as 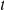 grows larger than a couple of years, this second term decays to zero. We model 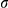 with Weibull survival functions, so that the times cases remain active in the population for are Weibull-distributed with distinct time scales for HIV-infected and uninfected TB cases.

**Figure 1:**
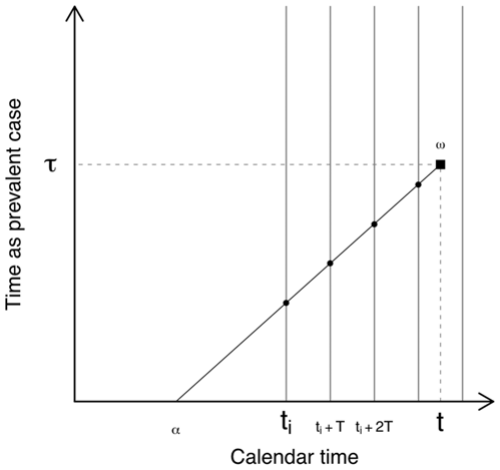
Modelling prevalence given incidence. An active TB case develops at 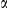, and remains active until 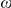. The probability that this case avoids death, self-cure or detection by routine services to remain active at 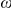 is 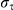. In the presence of 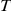-periodic active case-finding (vertical lines) beginning at time 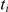, the case must also avoid detection by intersection with 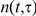 case-finding rounds (marked as filled circles), which occurs with probability 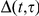.

Within this framework, PACF can be thought of as a series of impulsive hazards. Since we assume that the probability of being detected by PACF does not depend on 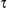, these are equivalent to fractional reductions in the density of active TB cases, 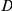, immediately after each round. We will call the fractional reduction the round efficiency, 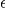. The round efficiency 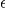 could be considered as representing the mean over some distribution of detection probabilities. One has
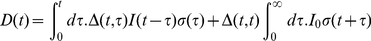
(22)where
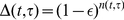
(23)for a round efficiency 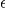, and where 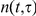 counts the number of rounds experienced by an active case at time 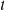 who has remained active and undetected for a duration 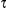. For an intervention with period 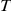, with initial round at time 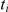,
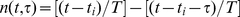
(24)where 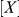 is the integer part of 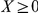 and 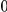 for 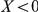. The meaning of Equation 22 can be visualised as shown in Figure 1.

We will make a proportional hazards assumption for the rate of detection, 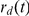, namely that 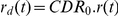 where 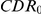 is the baseline case detection rate. This interpretation is justified as at equilibrium, with 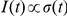, the detection probability for a case, given by parameter 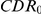, becomes equal to the WHO-defined CDR (the past year's notifications divided by the past year's total incidence).

The effects of subgroups of individuals who are less prone to detection, possibly in a way that correlates with their likelihood of being detected passively, can be conveniently modelled in this framework with appropriate mixture models for 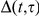.

### Parameter values

We assume 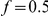, motivated by the reduced infectiousness of of smear-negative TB [20], and the increased likelihood of TB cases being smear-negative in HIV-infected individuals [19]. We model the distribution of ‘fast’ delays from infection to disease as exponential, with decay rate 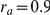 per year, derived by fitting an exponential to the delay distribution inferred in [15]. We model the survival functions as active, undetected TB, 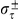, with Weibull distributions with typical durations in the presence of routine services taken as half the values specified in the absence of routine services in [21]. That is, we will take the survival shape and scale parameters for HIV-negative cases to be 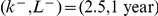 and for HIV-positive cases 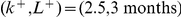. Our default proportion of TB cases due to recent transmission, 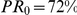, appropriate for high-burden settings [22], [23], and our default prevalence of HIV among incident TB cases is 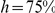, appropriate for high-HIV settings [1]. Our default baseline incidence will be 600 per 100,000 person-years.

For many of these parameters we explicitly consider the effects as their values change. This information is summarized in Table 1.

**Table 1:** Model parameters.

Parameter	Meaning	Value	Ref
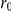	rate for recent activation	0.9/y	[15]
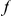	relative infectiousness HIV+ TB	0.5	[19], [21], [31]
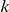	Weibull shape forduration active TB	2.5	guess
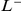	Weibull timescale (HIV−) forduration active TB	1 y†	[21], [32]
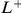	Weibull timescale (HIV+) forduration active TB	0.25 y†	[21], [33]
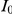	initial incidence TB disease	600. 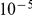 /y†	[1]
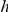	HIV prevalence inincident TB cases	75%†	[1]
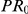	initial proportion TB diseaseincidence ‘recent’	72%†	[22]
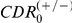	initial TB case-detectionrate (HIV+/−)	50%† *	[1]

† Default example: changes in parameter investigated.

*Only affects conclusions about numbers of cases found.

### Exploration of behaviour

Using asterisks to distinguish quantities under PACF from those without intervention, we calculate the total number of cases averted (TCA) as
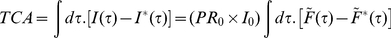
(25)All numerical computations were carried out using Euler-type methods with a time-step of 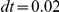 years, implemented in the R programming language [24]. To avoid interplay between the initial epidemic dynamics and the intervention, we began the intervention from the system at equilibrium. Round periods were chosen so that a whole number of rounds are performed during the intervention, and round-counts and responses were calculated incrementally before the final round. A comparison between the number of TB cases treated without intervention, and the intervention cost (measured in terms of treatment costs) added to the total number treated in this situation, was used as a very crude indicator of regions that might be cost-saving.

## Results

In this section we consider the behaviour of the model, as defined by Equations 14, 16, 17, 18, and 22.

**Figure 2:**
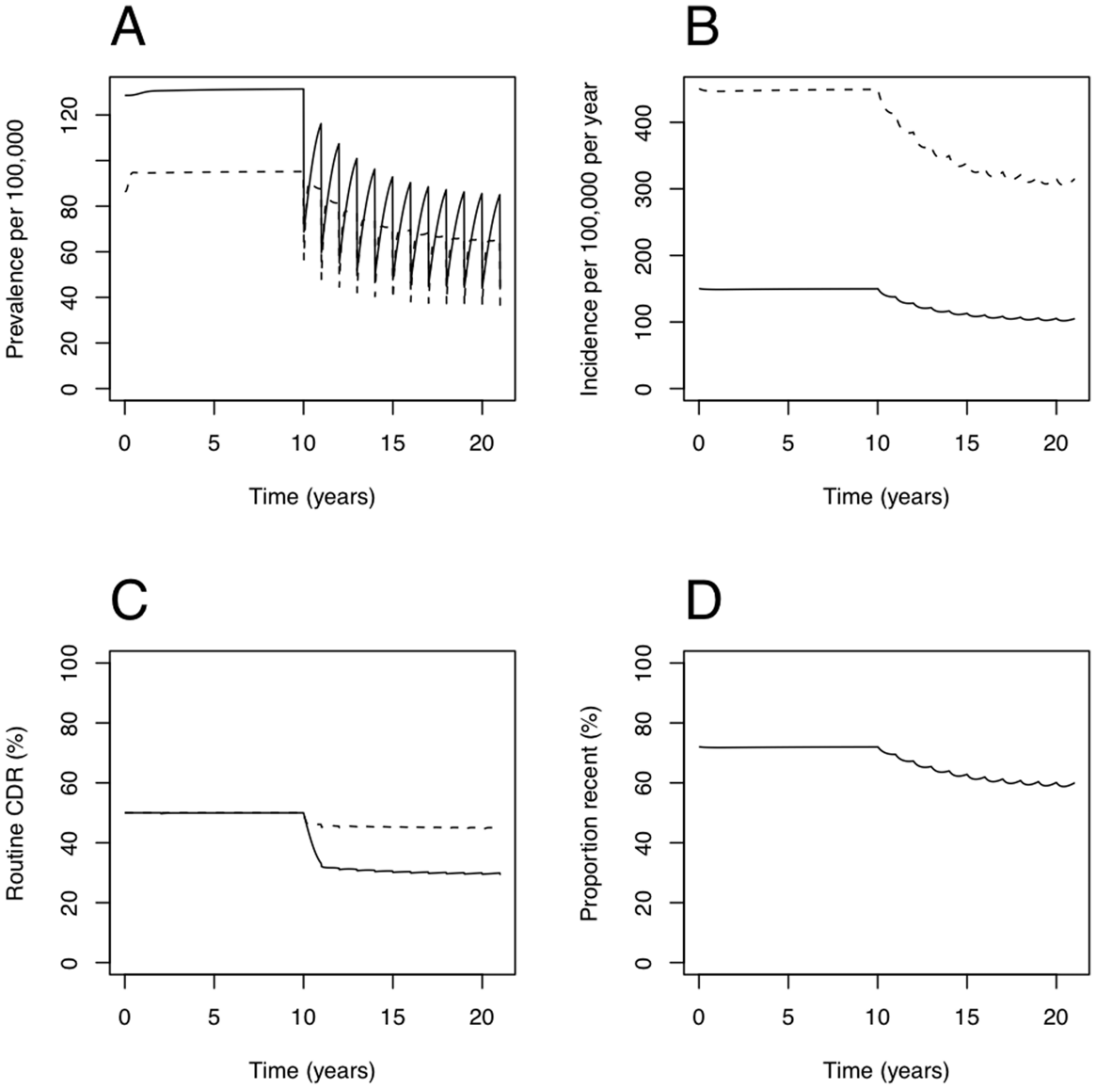
Example dynamics with yearly rounds from year 10. Panel A: prevalence of active undetected TB (dashed line for HIV-infected TB, solid line for HIV-uninfected TB; total population as denominator). Panel B: incidence of active TB (dashed line for HIV-infected TB, solid line for HIV-uninfected TB; total population as denominator). Panel C: case detection rate by routine services. Dashed line for HIV-infected TB, solid line for HIV-uninfected TB. WHO definition, i.e. the preceding year's detections divided by the preceding year's incidence. Panel D: the proportion of incident TB due to recent infection. All panels for the default parameters in Table 1, with 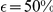, and 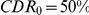.

Typical dynamics are shown in Figure 2: the prevalence of active TB is reduced by each round of case-finding (panel A), but begins to bounce back afterwards, slightly more rapidly for HIV-infected TB. Incidence also reduces (panel B), but in a smoother way, reflecting its dependence on a weighted sum over historical prevalence. Active case-finding works in competition with case detection by routine services, and so the CDR for routine services reduces once the intervention begins (panel C). This reduction is larger for HIV-uninfected TB than for HIV-infected TB, and model experimentation showed this to be a result of the shorter duration of HIV-infected TB. As prevalence reduces, the proportion of incident TB disease due to recent infection also reduces (panel D).

**Figure 3:**
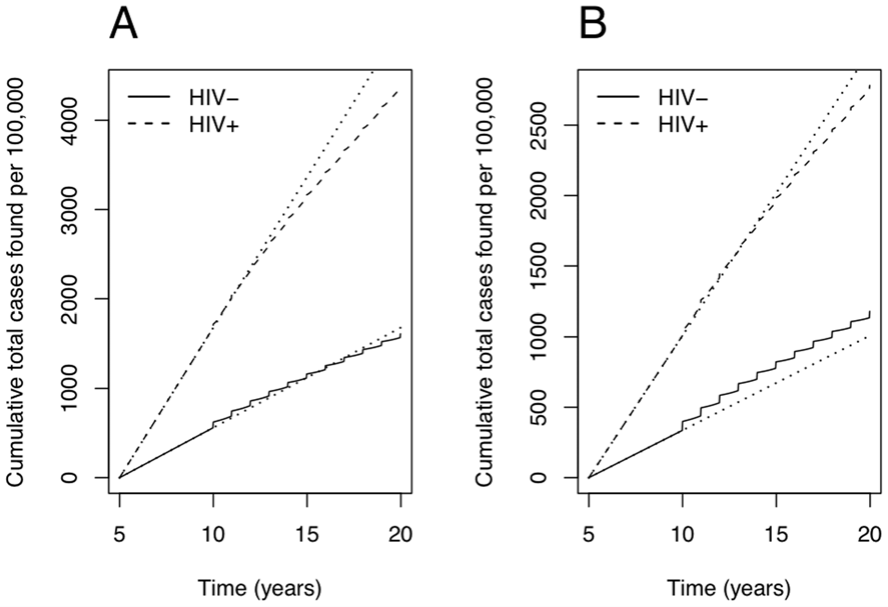
Cumulative cases found by both active and passive systems. Solid lines for HIV-uninfected TB, dashed lines for HIV-infected TB, and dotted lines for cumulative cases without interventions beginning at year 10. Both panels are for the default parameters of Table 1, with 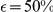. Panel A has 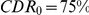, whereas panel B has case detection rates 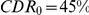. Fewer HIV-infected TB cases are found in total under the intervention in both panels. Fewer cases of HIV-uninfected TB are found in panel A, and more cases of HIV-uninfected TB in panel B.

Cumulative cases found by both routine services and the active case-finding rounds always increase initially (see Figure 3). However, since the intervention reduces incidence, by the end of 10 years one may either find more cases in total (solid line for HIV-uninfected TB in panel A of Figure 3), or fewer cases (solid line for HIV-uninfected TB in panel B of Figure 3, and the dashed line for HIV-infected TB in both panels).

**Figure 4:**
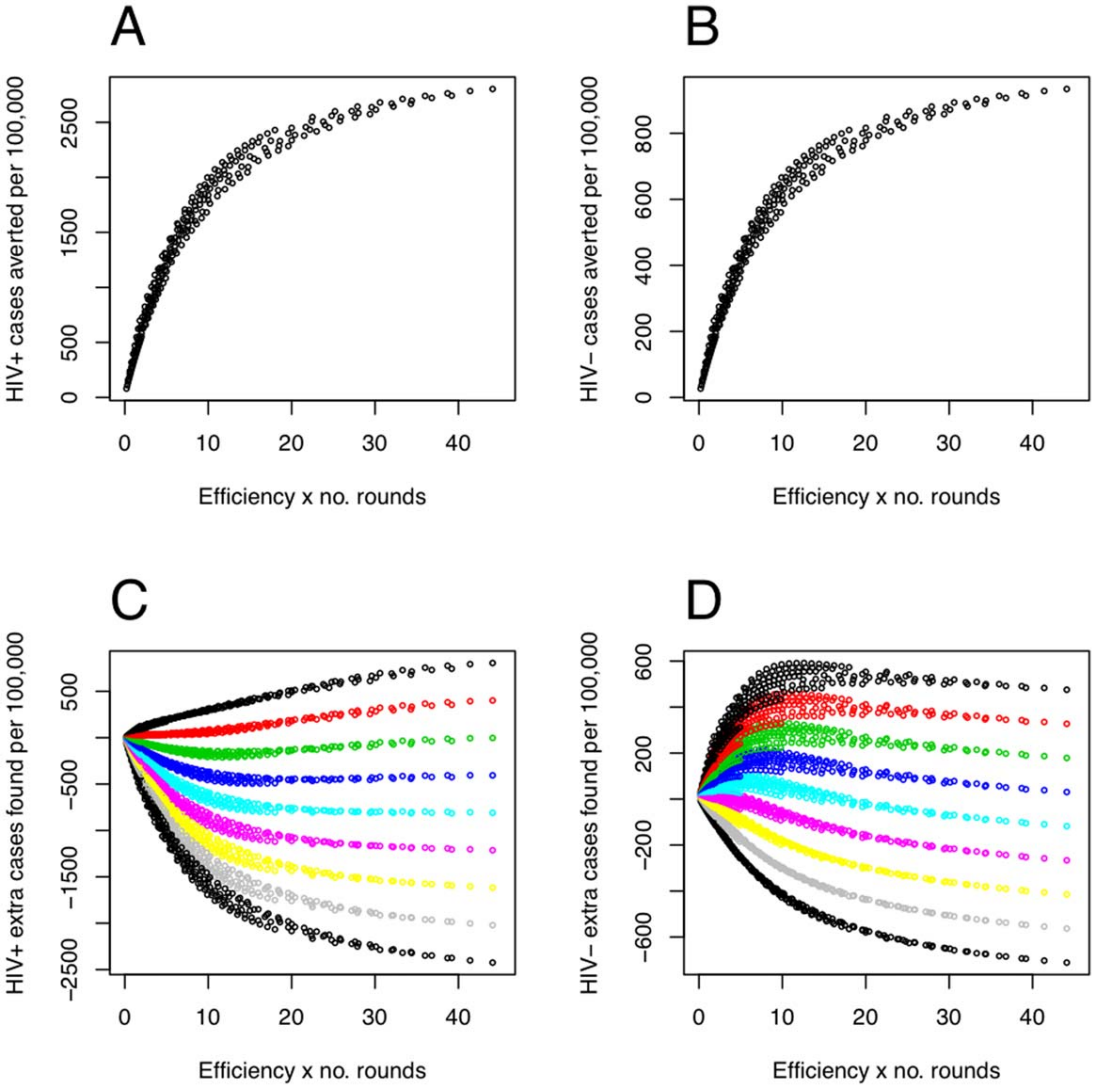
Cases found and averted for different round efficiencies, periods and initial case detection rates. Results from numerical model experiments plotted against the product of round efficiency and the number of rounds during the 10 year intervention period, in a population of 100,000. Panels A and B are the total HIV-infected and HIV-uninfected TB cases averted, respectively. Panels C and D are the total HIV-infected and HIV-uninfected TB cases found by both routine and active routes, respectively. Panels C and D have points colored by initial case detection rates; initial case detection rates do not affect cases averted. Realistic levels of achievement would be below 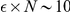, or a perfectly efficient round once a year. All panels have parameters other than those varied set to the default parameters of Table 1, although the overall level of incidence only affects the scales here. The round efficiencies, 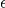, were chosen to vary from 10% to 90% in increments of 10%; and 28 values of the period 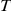 were investigated, chosen so that the number of rounds over the 10 years was an integer in the range 2 to 49.

Numerical experiments were carried out to investigate how different round efficiencies, 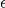, inter-round periods, 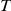, and initial case detection rates, 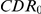, affected the number of cases found (by both routine and active routes), 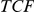, and the number of cases averted over a 10 year period (see Figure 4). The round efficiencies, 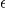, were chosen to vary from 10% to 90% in increments of 10%; and 28 values of the period 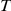 were investigated, chosen so that the number of rounds over the 10 years was an integer in the range 2 to 49. Plotting the results against 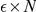 (where 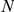 is the total number of rounds in this period) collapsed the data fairly well. The data in panels C and D are colored by 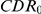, whereas the cases averted, 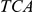, are independent of 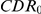. This fact can be seen directly from Equation 25. Moreover, since the HIV-uninfected and HIV-infected cases averted are driven by a shared difference in force-of-infection,
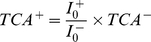
(26)The linearity of the dynamics implies that both 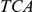 and 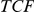 are proportional to the initial burden 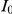:
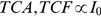
(27)Further, Equation 25 shows that
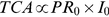
(28)since it is the recent part of the incidence that the intervention affects.

Interventions that could be realised in practice would likely have 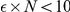, corresponding to 10 yearly, perfect case finding rounds. In this regime, the data in Figure 4, i.e. both 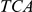 and 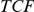, are roughly linear, i.e.
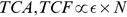
(29)


**Figure 5:**
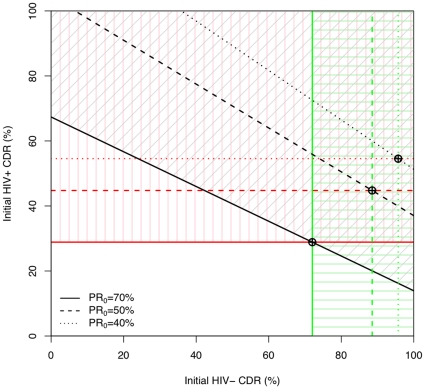
Approximate regions with fewer cases in total over the 10 years of the intervention. The red, vertically hatched regions are the critical initial case detection rates for HIV-infected TB, above which fewer HIV-infected TB cases are found by the combination of routine and active routes over 10 years. The green, horizontally hatched regions are the critical initial case detection rates for HIV-uninfected TB, above which fewer HIV-uninfected TB cases are found by the combination of routine and active routes over 10 years. The grey, diagonally hatched region indicates fewer total cases regardless of HIV-status. The default parameters of Table 1 are used but with 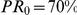. The HIV prevalence in incident TB affects the slope of the diagonal line, but the main factor that changes this picture is the initial proportion recent: results for 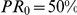 and 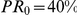 are indicated by dashed and dotted lines, respectively.

From inspection of panels C and D of Figure 4, one can see that it is primarily the initial CDR that determines whether fewer or more cases are found in total over the duration of the intervention, while 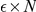, as a measure of effort, and 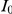, which scales the overall burden, determine the magnitude of this effect. Higher initial CDRs make it more likely that fewer cases will be found in total under the intervention. Figure 5 maps out the critical initial case detection rates for HIV-infected TB 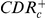 (red) above which fewer HIV-infected cases are found in total under the intervention, and similarly 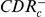 (green) is the rather higher value above which fewer HIV-uninfected TB cases will be found. The black line demarcates the region where fewer TB cases in total are expected to be found, regardless of HIV-status. It is not difficult to show that the slope of this lines is
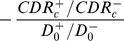
(30)so that this is strongly dependent on the HIV prevalence in incident TB cases. The values of 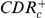 and 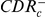 are not greatly affected by changed values of this HIV prevalence however, nor are they very sensitive to changed assumptions about the typical durations of HIV-infected and HIV-uninfected TB. As indicated in Figure 5 though, the proportion of incident cases initially due to recent infection, 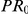, makes a large difference to these critical case detection rates.

**Figure 6:**
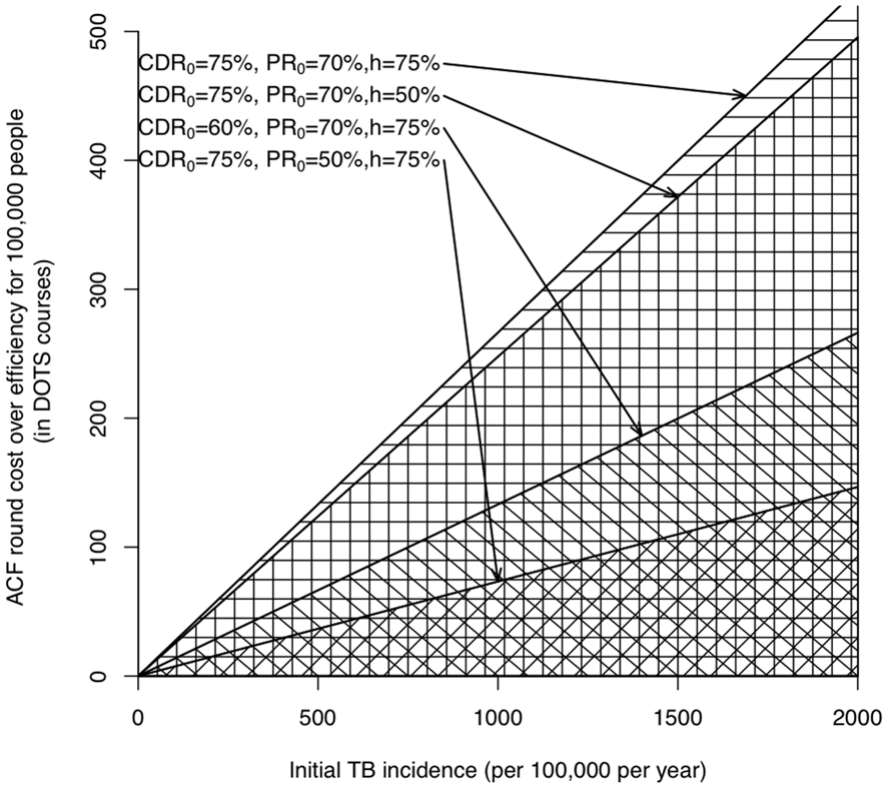
Approximate regions where intervention is cost-saving over 10 years. If the reduction in case load over the intervention is large enough to mean the treatments save (more than) pay for the case-finding rounds then the intervention saves the health system money. This happens for a high enough initial burden, and for a low enough cost per round relative to the cost of treatment (hatched regions). We have factored out efficiency to allow for approximate comparison between screening strategies of different efficiency. The HIV prevalence in incident TB, 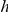, makes some difference; but the most influential factors in determining these regions are the initial case detection rates (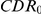) and the initial proportion recent (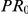).

For a high enough initial burden, there may be situations where the absolute size of the reduction in cases found compensates for the cost of implementing the case finding rounds through treatments avoided. Figure 6 plots the cost of the intervention in a population of 100,000 individuals divided by 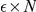 against the initial burden above which the intervention saves the health system money. Higher initial CDRs and higher proportions of incident disease due to recent infection favour cost-saving.

**Figure 7:**
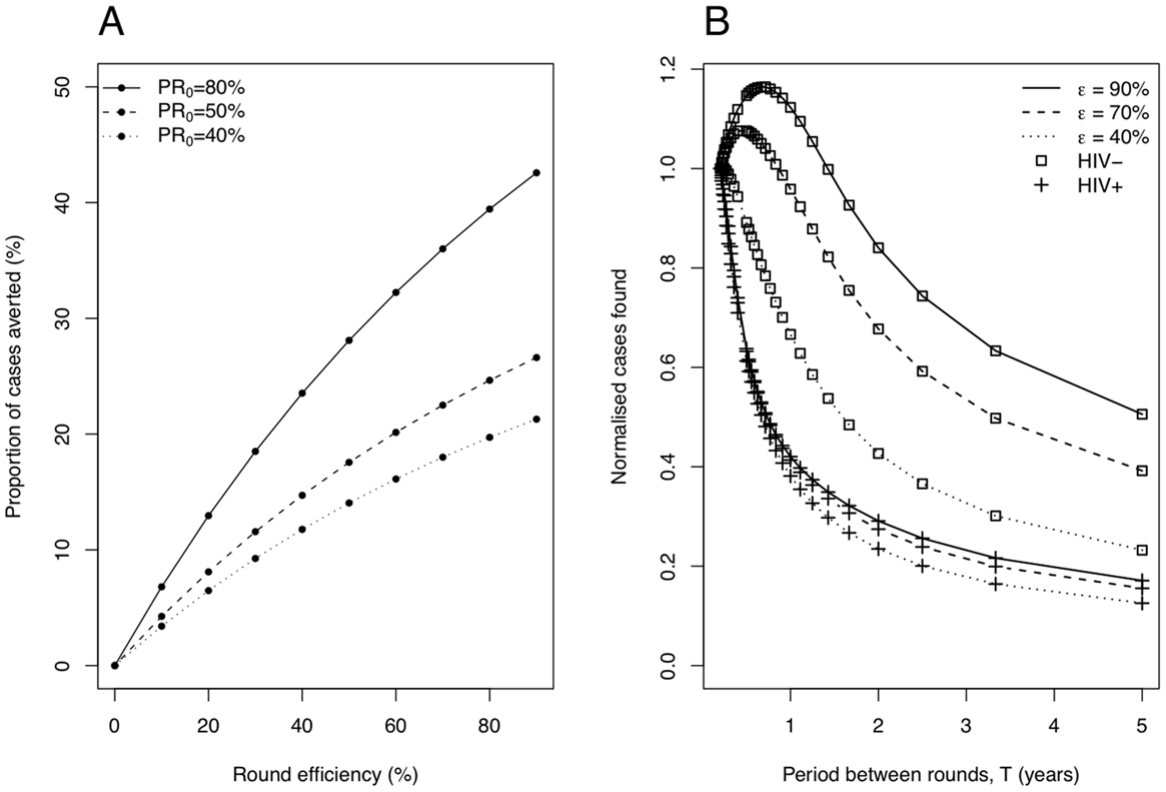
Proportion of cases averted for different round efficiencies and cases found at different periods. Panel A shows the total proportion of cases over a 10 year time frame averted by yearly case-finding rounds of different efficiency. Parameters are the default of Table 1, but with different initial proportions recent 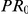. Panel B compares for rounds of different efficiency, 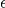, the influence of round frequency on the number of HIV-infected and HIV-uninfected TB cases found by the active route (normalized by the number of cases actively found at the shortest round period considered).

Panel A of Figure 7 factors out the initial burden to indicate the proportional number of cases one might expect to avert with 10 years of yearly case finding rounds of different efficiency. The initial proportion recent again makes a large difference to the achievable impact. Mindful that early diagnosis of cases through active case finding may also improve patient outcome, we consider the number of TB cases found by the active route at different round frequencies (panel B of Figure 7). There is a marked drop-off in numbers of HIV-infected TB cases found as rounds become less frequent, whereas at high round efficiencies the return of HIV-uninfected TB cases can increase from very frequent rounds that do not give the prevalent pool time to replenish, before falling again at low frequencies.

**Figure 8:**
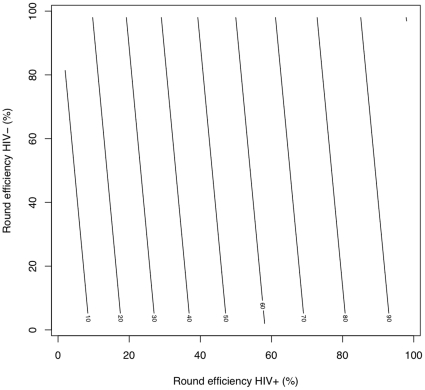
Contours of cases averted for different round efficiencies for HIV-infected and HIV-uninfected TB cases. These contours are the cases averted (as a percentage of the largest number averted) by yearly rounds with different efficiencies at finding HIV-infected and HIV-uninfected TB. Other parameters are the defaults of Table 1.

Finally, Figure 8 reports relative numbers of cases averted by scenarios with different case-finding round efficiencies for HIV-infected and uninfected TB cases. The numbers were computed for yearly rounds and the default parameters of Table 1. However, few of these make much difference, the picture being largely determined by 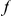 and the relative duration of HIV-uninfected and HIV-infected TB disease. Note that Equation 26 means this figure could equally refer to HIV-infected, HIV-uninfected or total TB cases averted. The contours indicate very little gain in cases averted by increasing the efficiency of HIV-positive TB case finding, compared with increasing the efficiency of HIV-negative TB case finding.

## Discussion

Traditional assessments of active case finding strategies have focused on cases found, and the number-needed-to-screen (NNS) to find one TB case. The NNS is a measure both of the efficiency of the screening strategy and the baseline prevalence of disease in a given population. If active case finding is used repeatedly, one expects the prevalence to fall, thus changing the NNS. ACF can be expected to reduce the passive notification rate (more strongly for HIV-uninfected TB), by acting in competition with it. However, while the overall number of cases found using active and passive systems combined will always increase initially, in the long-run introducing ACF may result in either an increase, or a decrease in the total number of cases found, depending on the efficiency of the passive system. Other things being equal, fewer cases are likely to be found in total as a result of repeated ACF when the routine system is functioning well and achieving a high CDR. This is essentially because for a poor passive system, fewer of the cases found by ACF would have been found otherwise, and are thus more likely to constitute extra cases for the health system. Our analysis also indicates that reductions in the cumulative HIV-infected TB case load are more likely than reductions in the HIV-uninfected TB case load, and that for situations where the proportion of TB incidence due to recent infection is below 40%, reductions in cases found become implausible or impossible.

The number of cases averted in a community by ACF increases with the overall burden and with the proportion of disease due to recent transmission. Every case averted represents a potential cost of treatment that the health system may be spared. On the other hand, finding TB cases and treating them both cost money. Nevertheless, if the cost of periodic screening is low enough, and the baseline burden high enough, our results suggest that ACF may provide a net cost saving over a 10 year period. While the cost of both treatment and case-finding vary by setting, it is their ratio that determines whether money is saved. This makes it natural to measure prices in terms of the cost of a DOTS treatment course, as we did in this analysis. In these units, Heller et al. estimated the price of an ACF round for a community of 100,000 people at around 33 [25], assuming 2% of the population sputum tested, with the price of smear microscopy twice its usual value to account for operational costs. If the case finding efficiency of a round based on smear microscopy were 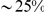, this would correspond to a value of around 120 on the 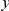-axis of Figure 6. As such, our results suggest that for baseline TB incidences above 500/100,000 per year, ACF would have the potential to be cost saving where the routine CDR and proportion of incidence due to recent infection are high enough, e.g. when both are around 70%. There appears to be scant data available in the literature on the operational cost of real mass screening strategies [26].

In comparing alternative screening strategies for a given community, we find that strategies with similar values for the product of detection efficiency and frequency of rounds can be expected to avert and find similar numbers of cases over the medium term. Thus, a screening strategy that is twice as expensive per round as another, so that it could only be used half as often for the same price, would have to be twice as efficient per round to achieve the same effect. A rule of thumb then, is to prefer the screening strategy with the lower cost per percentage point of round detection efficiency achieved. Practical limitations on round frequency may still favour high-efficiency, high-cost strategies when cheaper, less efficient alternatives could not be used frequently enough in practice to achieve a given effect.

TB has a different natural history, characteristic duration and clinical presentation depending on whether the individual is infected with HIV. There is therefore no reason to expect that the typical detection probabilities should not differ by HIV-status. Considering different case-finding efficiencies by HIV-status allows comparison of the impact, in terms of cases averted, of finding HIV-infected or HIV-uninfected TB cases. Reflecting the shorter duration and lower infectiousness of TB in HIV-infected individuals assumed in the model, the impact is far greater for each HIV-negative TB case found. This implies that we cannot rely on case-finding among HIV-infected individuals for the purposes of TB control at a population level. But this is not to say that finding TB among HIV-infected individuals is unimportant, or does not deserve particular attention. TB is more likely to be lethal in those with an HIV infection [21], [27], and their shorter duration as active cases means that they are more likely to fall into the gaps between rounds and escape detection. This means we should not rely on PACF to find those likely to die of HIV-related TB, but need more continuous and focused monitoring among those living with HIV infection. Our remarks on cost-effectiveness are necessarily simplistic at this level of generality; we aim rather at highlighting factors likely to be important and indicating mechanisms through which they act. A full cost-effectiveness analysis in a specific setting would allow even-handed inclusion of the benefits of deaths-averted from earlier diagnosis of HIV-infected TB cases, and infections averted from early removal of HIV-uninfected TB cases. The former would favour more frequent rounds, whereas the latter plays off against the discount rate to determine optimal frequency.

We have introduced a model of active case-finding for TB (in the presence of HIV) that is parametrized by a few, directly observed quantities, is conceptually transparent and explicit in its assumptions, and is simple enough to clarify some of the relationships between quantities of interest. As with all models, simplification entails limitations. The model is parametrized by specifying various key quantities at baseline, such as the proportion of disease that is due to recent infection, the incidence, and the routine case detection rate. As these are input parameters, they can be varied arbitrarily and independently in the model. In reality, one would not expect these baseline quantities to be independent, meaning that some combinations of parameter values would be unlikely to occur. For example, one would expect the proportion of incidence due to recent infection to correlate positively with burden in real populations [23], and possibly the case detection rate to correlate negatively with burden. This last correlation would limit the situations in which ACF could be expected to save money, but would influence a more comprehensive measure of cost-effect far less. We have also not considered here the possibility that the cost per round may have some dependence on disease burden, if more symptomatic individuals require more testing.

Other features of the natural history of TB were considered too uncertain to incorporate. Changes in infectiousness through time, and the extent to which infectiousness is prodromal, are important in determining the difficulty of control [28]. When considering ACF, a further distinction is required between clinical signs that may be detected by a case finding round, and symptoms that cause a patient to present at a clinic and become detected passively. The relationship between these is poorly understood, particularly through time, but data suggest that ACF appears to detect cases with less severe symptoms [29], [30]. The exact importance of this for TB transmission depends to what extent this represents early detection of cases who would later become more infectious, and remain so for some time before detection; and to what extent it represents detecting cases of permanently lower severity, and whether their presumably lower infectiousness off-sets their longer duration undetected. Another respect in which cases found actively may differ is in initial default rate: in South Africa, Den Boon et al. [30] observed higher initial default rates among cases found actively. The implications of this for mortality and transmission again depend on these aspects of TB natural history and patient behaviour.

Individuals are likely to be heterogeneous in their willingness to present for detection by ACF efforts, and may exhibit reducing willingness to participate in frequently repeated screening. If only a certain fraction of the population are accessible to ACF, then its impact in terms of cases averted will reduce by this fraction. The impact of ACF will be diminished further if those who do not present to ACF are the same individuals who do not present to routine services, and are thus responsible for a significant fraction of transmission.

Lastly, in order to avoid interactions between the dynamics of the epidemic and the effects of round timing, we considered intervening at equilibrium. The details, but not outline, of our conclusions are likely to be affected by the natural trend in TB incidence, and by changes in HIV prevalence and incidence risk ratio for TB given HIV-infection, which are all evolving. We would expect PACF in scenarios with an initial upward trend in TB incidence, *ceteris paribus*, to result in higher numbers of cases averted, and vice versa; though this need not be a strong effect if dominated by HIV-related TB.

A number of our assumptions could be classed as pessimistic, from the point of view of cost-efficacy. First, we have implicitly assumed that PACF, despite affecting force of infection, does not influence the prevalence of latent infection. In reality, the reduced risk of infection should reduce the latent prevalence and therefore yield more successful reductions in incidence. Secondly, our measure of the cost of disease only reflects the cost to the health service of treatment (if detected), and does not include any other negative externalities to society. Finally, it is also probable that regular screening rounds serve to raise community awareness of TB and its symptoms, and hence increase the chance of cases seeking care between rounds.

In summary, we have argued that if the burden of TB is high enough, ACF will be cost-effective. Indeed, in settings where the routine case detection rate is high as well as the TB incidence, ACF has the potential to be cost saving by reducing the total TB case load over a 10 year period. Conversely, in settings with poorly functioning health services, ACF is likely to increase the cumulative case load over the short and medium term, and thus care must be taken to ensure that treatment capacity is strengthened where necessary to meet demand. Finding more cases in total in a high burden settings with poor case detection does not mean that ACF will be less cost-effective there however: if the extra cases are treated, this extra cost will be buying them more positive health outcomes.

Our work repeatedly highlights the importance of the proportion of TB incidence due to recent infection as a key determinant of ACF effectiveness. If this proportion is less than about 40%, the impact of ACF is attenuated, and it is becomes very unlikely that enough cases will be averted over a 10 year period to reduce the cumulative case load. Whereas, if the majority of TB incidence in a community is due to recent infection, as seems to be the case where TB incidence is highest, repeated rounds of ACF have the capacity to avert a substantial proportion of TB cases.
